# The Additional Value of Tc 99m HMPAO White Blood Cell SPECT in the Evaluation of Bone and Soft Tissue Infections

**DOI:** 10.4274/MIRT.20.02

**Published:** 2011-04-01

**Authors:** Yasemin Şanlı, Zeynep Gözde Özkan, Seher Nilgün Ünal, Cüneyt Türkmen, Önder Kılıçoğlu

**Affiliations:** 1 Istanbul University Istanbul Medical Faculty, Department of Nuclear Medicine, Istanbul, Turkey; 2 Istanbul University Istanbul Medical Faculty, Department of Orthopedic Surgery and Traumatology, Istanbul, Turkey

**Keywords:** Infection, Tc 99m HMPAO, Tc 99m MDP, SPECT

## Abstract

**Objective: **The aim of this prospective study was to evaluate the additional value of Tc 99m HMPAO white blood cell (WBC) SPECT for bone and soft tissue infections.

**Materials and Methods:** Thirty-eight patients with suspected bone and joint infection were included in the study. Patients were assigned into 2 groups according to the presence of orthopedic implants. All patients had multiphase bone scan (BS) with Tc 99m methylene diphosphonate and WBC scintigraphy. The planar images of BS and WBC images were evaluated together. SPECT WBC images were evaluated separately.

**Results:** Group 1 had 30 patients including 12 patients with diabetic foot, 17 patients with suspected relapse of chronic osteomyelitis and 1 with septic arthritis. In 19 of 30 patients, BS and planar WBC images were similar with SPECT images in terms of final diagnosis. In the remaining 11 patients, planar BS+planar WBC images and SPECT WBC images were discordant. Group 2 included 8 patients with suspected infection of orthopedic implants. There was no difference between planar BS+planar WBC images and SPECT WBC in 6 of 8 patients. SPECT WBC images changed the diagnosis of 13 (34.2%) patients in the whole group. SPECT WBC images did not have any contribution in the evaluation of the 6 patients who had reactive changes.

**Conclusion:** SPECT images made significant contribution in discriminating soft tissue infection from osteomyelitis and improved diagnosis in terms of localization and the extent of disease.

**Conflict of interest:**None declared.

## INTRODUCTION

Although localizing the site of infection is important for planning treatment, the diagnosis and localization of bone infection represent a challenge for the physicians. The complementary use of scintigraphic and anatomic imaging modalities can overcome many of the limitations in the assessment of infection. Computed tomography (CT) accurately depicts sequestra, involucra, cloacae as well as soft tissue abscesses, foreign bodies, and fistulas (1,2). Despite of being very sensitive in detecting these bone changes, CT lacks specificity for bone infections which results in a high rate of false-positive findings (3,4). Meanwhile, planar images may fail to differentiate between osteitis accompanying soft tissue infection (STI) and osteomyelitis (OM). On the other hand, it is well established that SPECT/CT device provides accurate fusion of functional and anatomic imaging for the evaluation of endocrine, oncologic, and orthopedic events (5,6,7). However, SPECT/CT is not available in the vast majority of the nuclear medicine departments. 

The aim of our study is to evaluate the contribution of SPECT WBC as an adjunct to BS and planar WBC scan in patients being evaluated for the presence and localization of a skeletal infectious process.

## MATERIALS AND METHODS

Patients: Thirty-eight consecutive patients (27 males and 11 females; age range 10-93 years; mean age 51.5 years) with suspicion of having bone or orthopedic implant infection were prospectively evaluated in the present study. The scheme of the study was convenient with Declaration of Helsinki, 2008. According to the presence of orthopedic implants, the patients were divided into two groups. Group 1 included 30 patients; 12 patients with diabetic foot, 17 patients with suspected relapse of chronic OM, and 1 with septic arthritis. Group 2 included 8 patients with suspected infection of orthopedic implants; 4 with knee prostheses, 2 with tumor prostheses, and 2 with an implant in femur with suspected infection. Prosthesis was considered infected if in tissue cultures bacterial growth was present or in case of purulent finding at surgery. Prosthesis was considered uninfected in the absence of purulence at surgery and negative operative samples. In the cases in which no surgery was performed, the clinical outcome was monitored at least one year after the imaging procedure. On the basis of clinical parameters, at the end of follow up, the patients for whom antibiotic treatment was not required was considered as not infected. All patients were presented with clinical signs of infection and associated laboratory test results. 

**Radionuclide Studies:** All patients had BS with Tc 99m methylene diphosphonate (MDP) and WBC scan. The examinations were completed within 3 days at suitable intervals to avoid overlapping between the radionuclide studies. The findings of plain radiography, microbiologic culture, and long term clinical follow-up or surgical outcomes confirmed the final diagnosis. All of the images were obtained using a single head Siemens or dual-head ADAC Vertex gamma camera connected to a Pegasys computer (ADAC, Milpitas, CA) equipped with a low-energy, all purpose collimator and a 20% window centred on 140 keV.

**Multiphase Bone Scans:** BS was performed using 740 MBq (20 mCi) Tc 99m MDP. Immediately after injection, dynamic images of the related region (1 second frames) were obtained for 1 minute. Blood pool imaging was performed within 5 minutes after the tracer administration and delayed imaging 2-3 hours later.

**WBC scan:** Blood samples were collected for leukocyte labeling. 15 ml of blood was driven into the syringe which has 2 ml of ACD and 3 ml of HES. After 45 minutes, the plasma was separated and the remaining cellular part was centrifugated in 1000 cycles/min for 15 minutes. The plasma was separated again. Leukocyte fractions were labeled with Tc 99m HMPAO (Ceretec, Amersham) using a dose of 555 MBq (15 mCi) Tc 99m. After another centrifugation, the plasma was separated again and the remaining labeled leukocyte fractions were given to the patient. The labeling efficiency was 75-85%. The leukocytes were reinjected to each patient within 2.5 hours. The mean reinjected dose was 407-481 MBq (11-13 mCi). Tc 99m planar WBC images were acquired in anterior, posterior, and lateral or oblique projections at the 4th hour each for 10 minutes. SPECT WBC image was subsequently performed after the planar views. The protocol for SPECT WBC was as following: 360° acquisition, low-energy, all purpose collimators, 128x128 matrix, 30 steps, 40 s/frame. Images were reconstructed using Butterworth filtered backprojection (cutoff: 0.7; order: 5). Transverse, sagittal, and coronal slices were generated. Scintigraphic results were considered to be positive when one or more areas of uptake greater than background activity were identified.

**Image Analysis: **All planar and SPECT scintigraphic images were evaluated independently. Correlative images of X-ray, CT, or MRI were also regarded during these evaluations. 

**Image Interpretation:** The planar images of BS and WBC images were evaluated together. Meanwhile, SPECT WBC images were evaluated separately. The final diagnosis of infection was based on bacteriologic or histopathological criteria, examinations of surgical specimens, or clinical follow-up. 

**Multiphase bone scan: ** A bone scan was considered as positive in the presence of any focal or diffuse uptake in angiographic, blood pool, and the late images. 

**Tc 99m WBC Scan:** WBC scan was considered as positive when an abnormally localized activity, increased in intensity or in extension in comparison with the contralateral bone segment or the ipsilateral adjacent bone in the region of interest was observed. In addition, foci of suspected activity on the images were identified.

## RESULTS

The final diagnosis and a comparison of BS and planar WBC images and SPECT WBC images for groups 1 and 2 were summarized in Tables 1 and 2, respectively. 

**Group 1:** Group 1 had 30 patients, including 12 with diabetic foot in whom amputation was performed in 7 patients. Meanwhile, 17 patients had a suspicion of relapse of chronic OM (7 with past history of trauma, 6 had past history of surgery, and 4 had wound infection) and the remaining patient had septic arthritis. 

**Figure 1:**
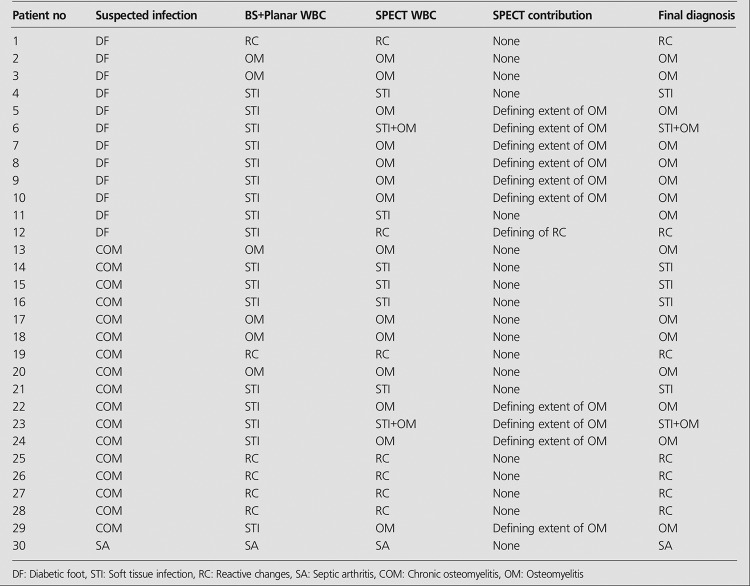
Tc 99m MDP bone scintigraphy of 30-y-old man in whom chronic osteomyelitis in left tibia was suspected. (a) Angiography, (b) blood pool, (c) late phase bone scintigraphy showed increased uptake in left tibia. (d) Tc 99m planar WBC scan showed increased uptake mainly in the soft tissue of left medial cruris. (e) SPECT WBC image localized abnormal uptake in soft tissue and left tibial region. Final diagnosis was found to be soft tissue infection and steomyelitis by biopsy

In 19 of 30 patients, BS and planar WBC images were concordant with SPECT WBC images for the final diagnosis and location of infection. SPECT WBC did not have any additional contribution to the management of these patients. Despite, BS and planar WBC images and SPECT WBC image results were found to be similar in 1 patient in whom the final diagnosis was false negative (Table 1, patient no: 11). The remaining 11 patients were discordant in terms of BS and planar WBC images and SPECT WBC images. In these patients, SPECT WBC clearly visualized the extent of the infection to deep tissues. One of these patients (Table 1, patient no: 12) had false positive result with planar images while SPECT WBC images were detected to be true negative according to final diagnosis. In the remaining 10 of 11 patients, the location of infection site was precisely defined and discriminated STI from bone infection with SPECT WBC (Figure 1). 

The final diagnosis was OM in 17 of 30 patients. Of these, 2 patients were presented with accompanying STI. Meanwhile, 5 patients had only STI and 1 patient had septic arthritis. The final diagnosis indicated no evidence of infection in 7 patients. 

**Figure 2:**
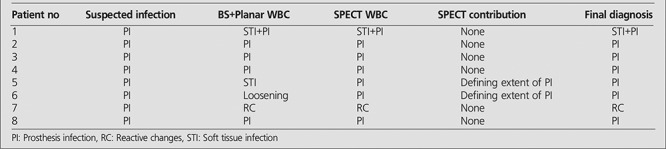
Tc 99m MDP bone scintigraphy of 22-y-old man in whom osteomyelitis of tumor prosthesis in left tibia was suspected. (a) Angiography, (b) blood pool, (c) late phase bone scintigraphy showed increased uptake in left tibia. (d) Tc 99m planar WBC scan showed faint uptake on left tibia. (e) SPECT WBC image clearly demonstrated increased uptake around tumor prosthesis. Final diagnosis was found to be prosthesis infection by surgery

**Group 2:** The results of BS and planar WBC images and SPECT WBC were similar in 6 of 8 patients with suspected infection of patients with orthopedic implants. In 2 patients, SPECT WBC changed the diagnosis. SPECT WBC images clearly defined prosthesis infection in 1 patient (Table 2, patient no: 5). The other patient had been operated 3 years ago for Ewing Sarcoma in left tibia. BS and planar WBC images showed loosening of tumor prosthesis instead of infection in prosthesis. However SPECT WBC images correctly localized WBC uptake in the tumor prosthesis (Figure 2).

SPECT WBC changed the diagnosis of 13 (34.2%) patients in the whole group. In addition, SPECT WBC did not make any contribution to the evaluation of the patients with reactive changes. 

The sensitivity, specificity, positive predictive value, negative predictive value, and accuracy rates for planar and SPECT images in all patients and also in groups 1 and 2 are mentioned in Table 3. Meanwhile, results of BS and planar WBC images and SPECT WBC images in relation with the type and location of infection in Groups 1 and 2 are given in Table 4 and 5.

## DISCUSSION

Diagnosis of OM with noninvasive techniques can be difficult particularly with previous trauma, surgery, or infection. Planar WBC has been used widely for the diagnosis of these complicated OM (8,9,10) . However, the value of these planar scintigraphic images is limited due to their low image resolution. This difficulty is more pronounced when adjacent STI is adjoined to the bone infection.

Up to date, some studies have been performed for the diagnosis of orthopedic infections with SPECT and SPECT/CT. Similar to our study, Weon et al. evaluated incremental value of additional SPECT images by using Tc 99m HMPAO leukocyte scans to evaluate bone infection and reported the overall sensitivity of the Tc 99m HMPAO leukocyte scan with SPECT to detect bone infection as 92%, with a specificity rate of 85% . In our study, we found that overall sensitivity and specificity of the BS and planar WBC scan to detect infection site was 45.8% and 92.8%, respectively and these rates increased to 95.8% and 100% with the inclusion of SPECT WBC scan. Thus, it is rational to consider that SPECT WBC scan significantly contributes to BS and planar WBC scan that is currently under use. Meanwhile in the former study it was detected that the faint and diffuse activity in the planar images caused false negative results, but higher lesion to background ratio with the SPECT changed the diagnosis. The greater contrast provided by SPECT allows more precise localization of sites of leukocyte accumulation. The use of SPECT has been proposed to avoid obtaining two scans (BS and planar WBC scan) and accurate delineation of the extent of the infectious process even superficial or in deep tissues. Similar to this observation, in12 of 38 patients in our study, false negative image interpretation changed to true positive in terms of OM with the inclusion of SPECT WBC images and false positive image interpretation of one patient changed to true negative with SPECT WBC images.

We evaluated the value of SPECT WBC in diabetic foot infections in the present study and found that SPECT WBC findings changed 6 of 12 patients (50%) with STI to the diagnosis of OM. There was only one false positive patient (Table 1, patient no: 12) with BS and planar WBC images in which the lesion was in the forefoot and the patient had traumatic fracture at the same place. SPECT WBC images clearly demonstrated that there was no focal pathological uptake. Thus, the faint uptake in this area was a result of previous trauma.

On the other hand, Bar-Shalom et al. evaluated SPECT/CT using Ga 67 and in 111 labeled leukocyte scintigraphy for diagnosis of infection, as compared with planar and leukocyte SPECT. The authors reported that, SPECT/CT provided additional information for the diagnosis and localization of infection in 48% patients . This high contribution of SPECT/CT is probably due to the heterogeneity of patients studied including infections from other organs such as brain, chest, and abdomen in which CT may be more helpful. In another aspect, the rate of 100% specificity in our study questions the need for additional SPECT/CT for the evaluation of patients with osteomyelitis and STI. In a similar fashion, Horger et al. showed that sensitivity was identical for SPECT and SPECT/CT whereas specificity was higher for SPECT/CT (78% versus 89%) . However our specificity with only SPECT images is higher from the study by Horger et al. It is worth mentioning that the small number of patients in Group 2 limits the value of our technique in patients with orthopedic implant concomitantly having infection. In another study, Filippi et al. has studied 28 patients with bone and joint infections and found that SPECT/CT added a significant contribution in 10 of 28 (35.7%) patients . In our study this ratio was found 34.2% which is in accordance with other studies (14,15).

SPECT/CT hybrid systems allow the acquisition of functional and morphologic images in a single session. Low dose CT improves SPECT image quality. However, in the case of a patient with prosthesis who is studied with low dose CT, metal artifacts due to prosthetic implants can be confusing. Meanwhile, SPECT/CT is not available in many nuclear medicine departments. In addition, Strobel et al. has compared focal bone lesions with planar bone scan with SPECT and SPECT/CT. Their results showed that the difference in diagnostic performance from planar scintigraphy to SPECT and SPECT/CT was not significant (16). 

FDG PET has demonstrated greatest utility in bone infections. Activated inflammatory cells like malignant cells, metabolize glucose as a source of energy. In stimulated state, inflammatory cells express high concentrations of glucose transporters that facilitate the movement of FDG through the cell (1^7^).

FDG PET is promising in previously documented chronic osteomyelitis with suspected recurrence and symptoms of osteomyelitis with more than 6 weeks. In contrast to other nuclear medicine studies, FDG PET has higher resolution and can distinguish STI from OM (17). FDG PET imaging can also differentiate between Charcot neuropathy, osteomyelitis, and soft tissue infection (18). 

FDG PET/CTimaging may be limited in the presence of metallic artifacts such as those caused by prostheses adjacent to the sites of infection. FDG PET imaging showed the same sensitivity as that of combined WBC /bone marrow scintigraphy for detecting infection, but the specificity was greater for the latter method (17). 

Avidity of inflammatory cells for F 18 FDG has led to the concept of ex vivo labeling of leukocytes with F 18 FDG. Infections can be imaged with F 18 FDG–WBC PET/CT. In patients suspected of having focal infection, F 18 FDG PET demonstrates sensitivity similar to that of İn 111 labeled leukocytes, but it has lower specificity due to false-positive signal in tumor and postoperative changes (19).

Major difference between F 18 FDG–WBC PET/CT and F 18 FDG PET/CT relates to the cellular types involved in the signal detected in infected sites. F 18 FDG reveals different types of inflammatory cells, including macrophages, residing within the lesions, whereas F 18 FDG–WBC essentially reveals active diapedesis of granulocytes through chemotactic processes. Results show that F 18 FDG–WBC PET/CT is a promising technique with a high sensitivity and specificity and has major advantages over the more conventional nuclear medicine and radiologic methods (19). Its potential role as a diagnostic instrument in infection imaging merits further investigation in larger prospective series.

## CONCLUSION

SPECT WBC images had a significant contribution in discriminating soft tissue infection from osteomyelitis. SPECT WBC improves diagnosis, localization, and the extent of disease and increases the specificity of the imaging modality. We believe that SPECT WBC may be useful when planar images are positive for infection but equivocal for localization of the infection site. However, SPECT WBC does not contribute to the evaluation of patients in the case of negative planar scans. 

## Figures and Tables

**Table 1:**
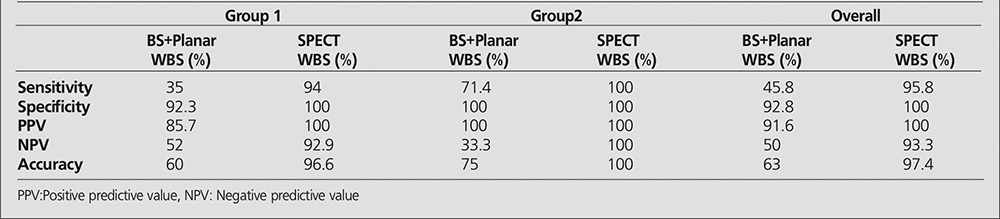
Results of multiphase bone scintigraphy+planar WBC and SPECT WBC findings in Group 1

**Table 2:**
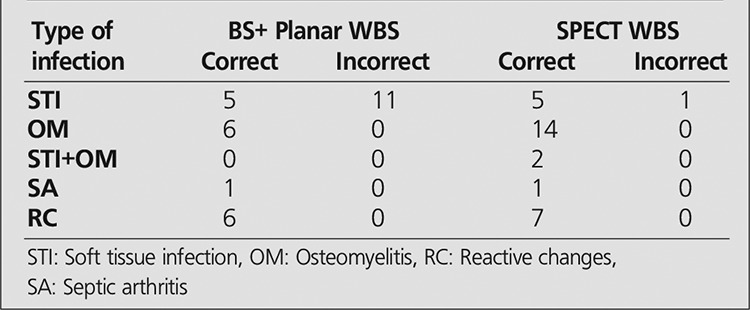
Results of BS+planar WBC and SPECT WBC findings in Group 2

**Table 3:**
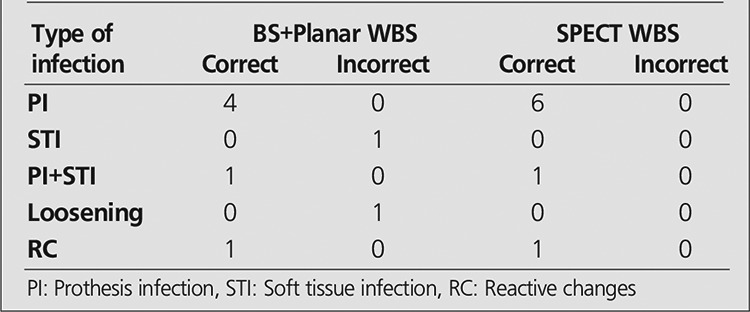
Sensitivity, specifity, PPV, NPV, and accuracy rates in Group 1, 2 and overall patients

**Table 4:**
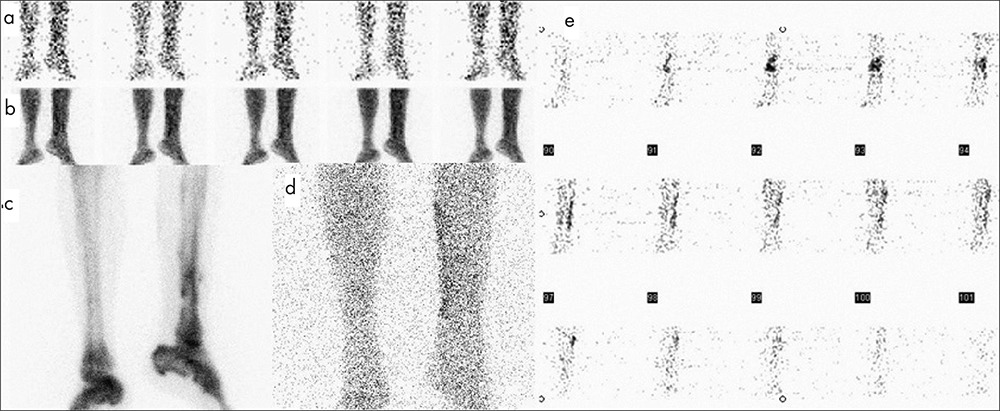
Results of BS+planar WBC scintigraphy and SPECT WBCscan regarding type and location of infection in Group 1

**Table 5:**
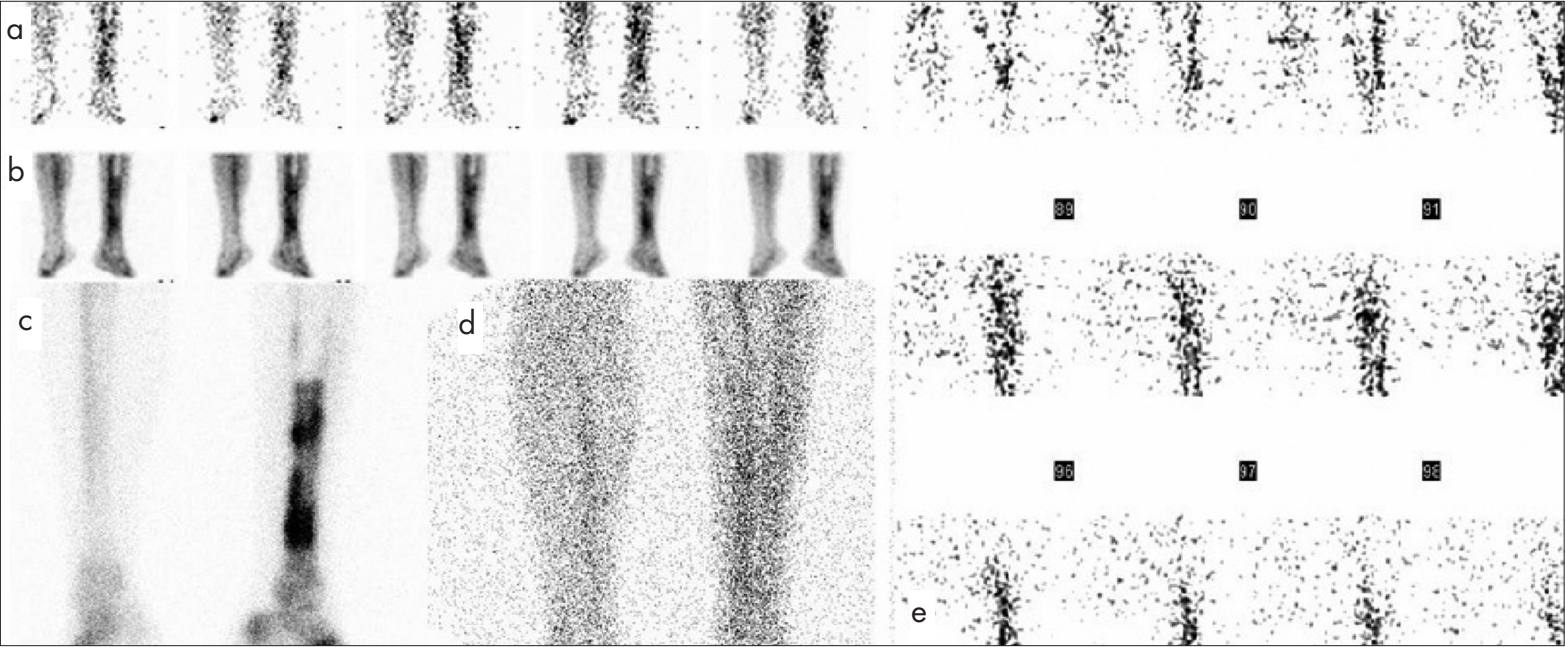
Results of 3P+planar WBC scintigraphy and SPECT WBCscan regarding type and location of infection in Group 2
